# Functional Analysis of Promoters, mRNA Cleavage, and mRNA Secondary Structure on *esxB-esxA* in *Mycolicibacterium smegmatis*

**DOI:** 10.3390/pathogens13121041

**Published:** 2024-11-27

**Authors:** Ryan G. Peters, Jessica M. Kelly, Sarah Bibeau, Ying Zhou, Scarlet S. Shell

**Affiliations:** Department of Biology and Biotechnology, Worcester Polytechnic Institute, Worcester, MA 01609, USA; ryanpeters.science@gmail.com (R.G.P.); jessknr99@gmail.com (J.M.K.); sbibeau2017@gmail.com (S.B.); yzhou9@wpi.edu (Y.Z.)

**Keywords:** mycobacteria, *Mycobacterium smegmatis*, ESX-1, EsxA, EsxB, mRNA cleavage, mRNA processing, mRNA stability, 5′ UTR, transcription start site, transcriptional regulation, promoter, type VII secretion system

## Abstract

The ESX-1 secretion system is critical for the virulence of *Mycobacterium tuberculosis* as well as for conjugation in the saprophytic model *Mycolicibacterium smegmatis*. EsxB (CFP-10) and EsxA (ESAT-6) are secreted effectors required for the function of ESX-1 systems. While some transcription factors regulating the expression of *esxB* and *esxA* have been identified, little work has addressed their promoter structures or other determinants of their expression. Here, we defined two promoters, one located two genes upstream of *esxB* and one located immediately upstream, that contribute substantially to the expression of *esxB* and *esxA*. We also defined an mRNA cleavage site within the *esxB* 5′ untranslated region (UTR) and found that a single-nucleotide substitution reprogramed the position of this cleavage event without impacting *esxB*-*esxA* transcript abundance. We furthermore investigated the impact of a double stem-loop structure in the *esxB* 5′ UTR and found that it does not confer stability on a reporter gene transcript. Consistent with this, there was no detectable correlation between mRNA half-life and secondary structure near the 5′ ends of 5′ UTRs on a transcriptome-wide basis. Collectively, these data shed light on the determinants of *esxB-esxA* expression in *M. smegmatis* as well as provide broader insight into the determinants of mRNA cleavage in mycobacteria and the relationship between 5′ UTR secondary structure and mRNA stability.

## 1. Introduction

*Mycobacterium tuberculosis* kills more people each year than any other infectious pathogen besides SARS-CoV-2 [1]. In 2022 alone, approximately 1.3 million people died from tuberculosis and over seven million people fell ill [1]. The success of *M. tuberculosis* comes in part from its ability to survive within, and ultimately escape, host macrophages. The ESX-1 type VII secretion system is critical for *M. tuberculosis*’s ability to block phagosomal maturation and subsequently rupture the phagosomal membrane to escape into the cytosol (reviewed in [2,3,4,5]). Understanding the mechanisms regulating the expression of ESX-1 components is therefore crucial to gaining a better understanding of *M. tuberculosis* pathogenesis. Interestingly, ESX-1 is conserved in non-pathogenic mycobacteria as well, and in the saprophytic model *M. smegmatis*, it is required for conjugation [6,7].

ESX-1 is one of at least five type VII secretion systems encoded by *M. tuberculosis*. These systems contain a mixture of ESX conserved components (Ecc), ESX-specific proteins (Esp), and other proteins including PE/PPE proteins, small secreted Esx proteins, and MycP (reviewed in [5]). Many of the ESX-1 components are transcribed from genes located in a single genetic locus, part of which is termed Region of Difference 1 (RD1) due to its loss from the *M. bovis* bacille Calmette–Guérin (BCG) vaccine strain [8,9]. RD1 includes the genes encoding EsxA and EsxB (also called ESAT-6 and CFP-10, respectively), which are some of the first-discovered and most-studied components of the ESX-1 system. EsxA and EsxB are both secreted virulence factors and are additionally required for the secretion of any substrates by the ESX-1 system [10]. EsxA and EsxB form a heterodimeric complex and may also have activities on their own (reviewed in [2,3,4,5]). They interact with host proteins to subvert phagolysosomal fusion, thus permitting survival and even growth of *M. tuberculosis* within phagosomes (reviewed in [2,3,4,5]). They also contribute to phagosomal membrane disruption and thus the access of *M. tuberculosis* to the host cell cytoplasm, with numerous consequences for bacterial nutrition, host cell fate, and immune activation and subversion (reviewed in [2,3,4,5]).

Defining the mechanisms underlying the regulation of virulence factors is important for understanding how mycobacterial pathogens respond to the microenvironments and perturbations that they encounter during infection. The expression of *esxA* and *esxB* appears to be regulated at least in part by the transcription factor WhiB6 [11,12], which is itself regulated by other transcription factors including PhoP [12]. Various transcription start sites (TSSs) have been mapped within the genomic region containing *esxA* and *esxB* by transcriptome-wide TSS-mapping studies [13,14,15,16], but the relative contributions of each of these to the expression of genes in the locus have not been defined.

The genes *esxB* and *esxA* have been predicted to be transcribed as part of an operon in *M. tuberculosis*, with early work predicting a two-gene operon [17]. However, global mappings of transcription start sites (TSSs) in *M. tuberculosis* produced conflicting results, with one study suggesting that *esxB* and *esxA* are transcribed together along with the two upstream genes *PE35* and *PPE68* [14,15] and another suggesting that *esxB* and *esxA* each have their own promoters [13]. Further complicating this situation, one of the 5′ ends upstream of *esxB* that was annotated as a TSS in one study was annotated as an mRNA cleavage site in another [13,14,15].

The non-pathogenic model *M. smegmatis* encodes orthologs of PE35, PPE68, EsxB, and EsxA with amino acid sequence identities of 52.2%, 45.4%, 62.2%, and 71.6% compared to their *M. tuberculosis* counterparts, respectively (data obtained from https://orca1.tamu.edu/mad/orthologs/pages/Rv3872.html, accessed on 11 November 2024). As a free-living organism that does not infect other cells, *M. smegmatis* appears to use its ESX-1 system primarily for performing and regulating the process of distributive conjugal transfer (DCT). DCT is a horizontal gene transfer modality that is mechanistically distinct from conjugation in better-studied bacteria such as *E. coli* [18]. DCT in *M. smegmatis* requires genetically distinct donor and recipient strains. Disruptions in a number of ESX-1 genes, including the PE35 and PPE68 orthologs, caused a donor strain to have dramatically increased rates of DCT [7]. This phenotype was partially complemented by the *M. tuberculosis* orthologs of these genes [7], indicating the conservation of the mechanistic functions of ESX-1 components between the two species, despite the different roles the systems play. Surprisingly, disruptions of several ESX-1 genes, including the PE35 ortholog, caused an *M. smegmatis* DCT recipient strain to have greatly reduced DCT efficiency, indicating that ESX-1 represses DCT in donor cells, yet is required for DCT in recipient cells [6]. This appears to be due at least in part to cross-talk between the ESX-1 system and another type VII secretion system, ESX-4 [19]. It is notable that while *M. tuberculosis* does not appear to undergo DCT and rarely encounters other bacteria, DCT may have roles in generating genetic diversity and in the exchange of drug resistance in mycobacterial pathogens that have environmental niches and/or encounter other bacteria during infection, such as *M. abscessus*.

Given the importance of ESX-1 systems in various mycobacteria, we sought to better understand the mechanisms by which expression of the ESX-1 effectors EsxA and EsxB is controlled. In *M. smegmatis*, our previous transcriptome-wide study suggested the presence of a TSS upstream of *PE35*; a TSS between *PPE68* and *esxB*; a major cleavage site between *PPE68* and *esxB*; and two TSSs within *esxB* [20]. Here, we tested the roles of two predicted promoters, the mRNA cleavage event, and the secondary structure in the 5′ untranslated region (UTR) of the *esxB* transcript. We present a model of the determinants of expression of the *PE35-PPE68-esxB-esxA* locus involving at least two promoters and show that the *esxB* and *esxA* transcripts have longer half-lives than the *PE35-PPE68* transcript but that this does not seem to be explained by 5′ UTR structure. Additionally, we show that the position of the major RNA cleavage site between *PPE68* and *esxB* can be reprogrammed by a single-nucleotide substitution.

## 2. Materials and Methods

### 2.1. Strains and Culture Conditions

**Figure 1:**
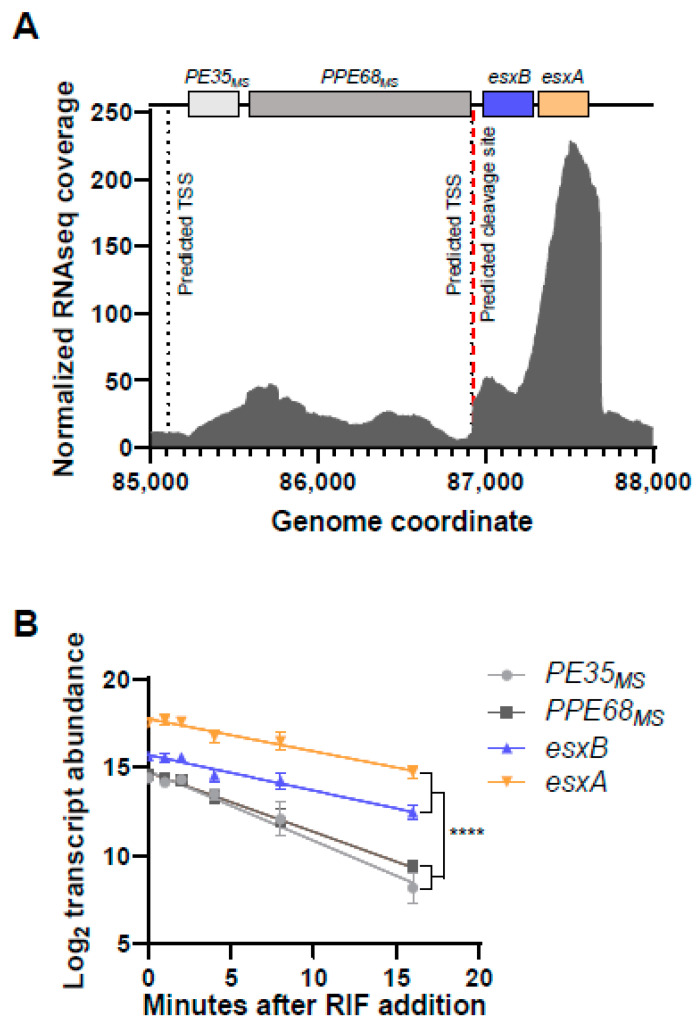
The *esxB* and *esxA* transcripts have higher abundance and longer half-lives than the transcripts encoded upstream. (**A**) RNAseq coverage for the *M. smegmatis* region encompassing *PE35_MS_*, *PPE68_MS_*, *esxB*, and *esxA* (*msmeg_0063-0066*). The data are from [15]. The genes of interest are shown to scale above the transcript abundance plot. Two previously mapped transcription start sites (TSSs) and an RNA cleavage site are shown with dashed black and red lines, respectively [20]. The second TSS appears at nearly the same position as the cleavage site in this graphic because they are only 4 nt apart. The coordinates are from NC.008596.1. (**B**) The degradation rates of the indicated transcripts were assessed by measuring transcript abundance following the addition of rifampicin (RIF) to block transcription. The slopes of the decay curves were compared by linear regression. The slopes of *PE35_MS_* and *PPE68_MS_* were steeper than the slopes of *esxB* and *esxA* (**** *p* < 0.0001 for each pairwise comparison between the two groups), while the slopes of *PE35_MS_* and *PPE68_MS_* were not significantly different from each other nor were the slopes of *esxB* and *esxA* (*p* > 0.05 for each pairwise comparison).

**Figure 2:**
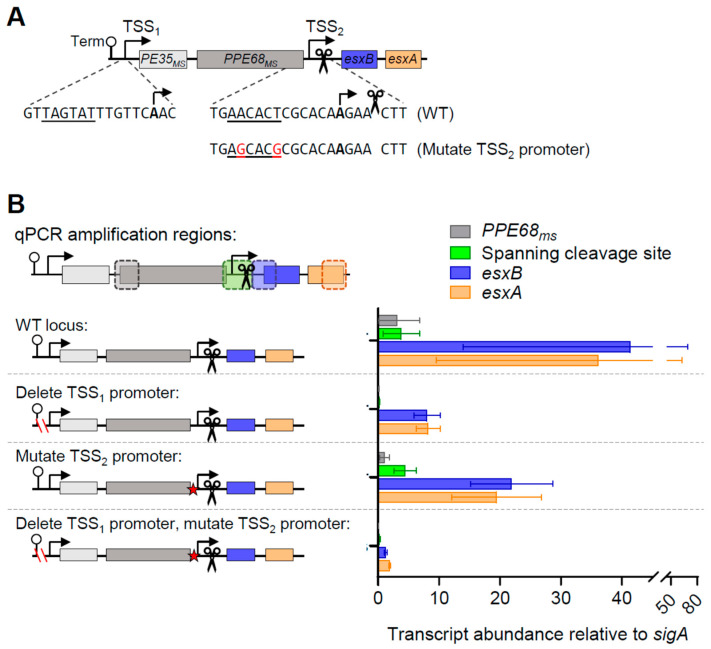
A second promoter is present between *PPE68_ms_* and *esxB*. (**A**) A schematic of a cassette cloned into an episomal plasmid lacking a promoter to test the expression of ESX-1 genes. The cassette contains the tsynA transcriptional terminator [21] to block transcription from spurious promoter sequences in the plasmid backbone. TSS_1_ and TSS_2_ were previously mapped in [20]. A previously mapped cleavage site (Martini et al. 2019) is indicated with scissors. The elements in the construct are not drawn to scale. The mutations to the putative promoter for TSS_2_ are shown. (**B**) The plasmids harboring variations of the cassette shown in A were transformed into an *M. smegmatis* strain with a deletion of *msmeg_0062* (*eccC1b*) through *msmeg_0066* (*esxA*). RNA was harvested from biological triplicate cultures in log phase, and qPCR was used to measure the expression of three genes and a region spanning the cleavage site. The data are representative of three independent experiments.

**Figure 3:**
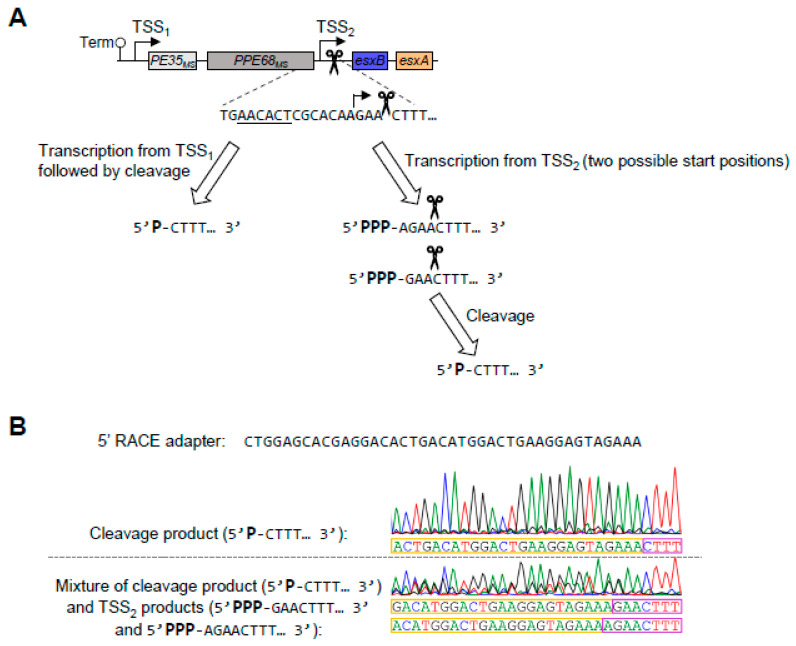
The promoter between *PPE68_ms_* and *esxB* initiates transcription 3–4 nucleotides upstream of a major cleavage site. (**A**) A schematic of the RNA 5′ ends between *PPE68_ms_* and *esxB* that arise from a previously mapped cleavage site [20] and a promoter defined in Figure 2 and here. (**B**) An example of Sanger sequencing traces from 5′ RACE used to map 5′ ends in the region between *PPE68_ms_* and *esxB*. The 5′ RACE adapter sequence is shown and indicated with yellow boxes. The RNA sequences being mapped are boxed in purple. The top trace is an example of a clean trace that primarily reflects the previously mapped RNA cleavage site. The bottom trace is an example of a mixed-peak trace where three sequences can be seen: that arising from the cleaved RNA and those arising from two transcripts made from TSS_2_, which has two possible start positions. The sequencing traces are representative of at least three separately sequenced gel bands.

**Figure 4:**
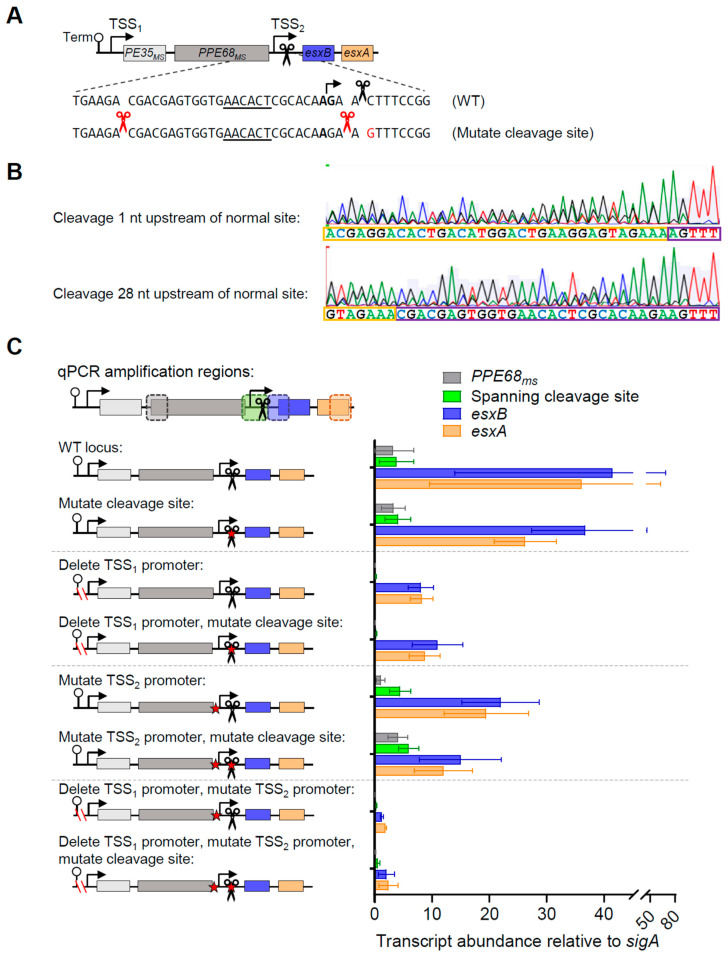
mRNA cleavage can be reprogrammed by a point mutation but does not appear to affect the expression of *PPE68_ms_*, *esxB*, and *esxA*. (**A**) The constructs shown in Figure 2A were subject to a point mutation immediately downstream of the cleavage site (C→G, red font). (**B**) 5′ RACE was used to map RNA 5′ ends in the vicinity of the cleavage site. Shown are two traces from a strain harboring a construct with the cleavage site C→G mutation. While both traces contain mixed peaks, in the upper trace, a sequence beginning 1 nt upstream of the normal cleaved 5′ end can be seen, while in the lower trace, a sequence beginning 28 nt upstream of the normal cleaved 5′ end can be seen. The sequencing traces are representative of at least three separately sequenced gel bands. (**C**) The data from Figure 2 are shown with the addition of constructs with a point mutation downstream of the cleavage site. The data are representative of two independent experiments, each performed with biological triplicate cultures.

**Figure 5:**
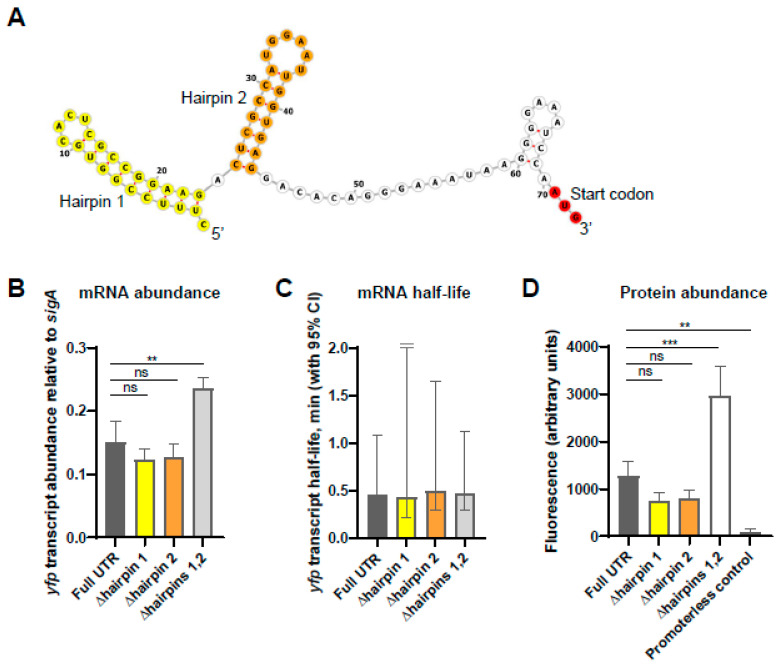
The secondary structure in the *esxB* 5′ UTR does not impact transcript half-life. Constructs were made, in which variants of the *esxB* 5′ UTR were linked to the coding sequence of *yfp* in integrating plasmids and transformed into wildtype *M. smegmatis*. (**A**) The predicted secondary structure of the *esxB* 5′ UTR and start codon (red) following cleavage. The hairpin structures are indicated in yellow and orange. This is designated “Full UTR” in subsequent experiments. The variants lacked one or both hairpins. The graphic was made with the ViennaRNA Web Services tool *forna*. (**B**) The abundance of the *yfp* transcript fused to the indicated *esxB* 5′ UTR variants was measured by quantitative PCR and expressed relative to the housekeeping gene *sigA*. The mean and SD of triplicate samples are shown. The means were compared by ANOVA followed by Dunnett’s multiple comparisons test to compare “Full UTR” to each of the variants. **, *p* < 0.01; ns, *p* > 0.05. (**C**) Half-lives of the *yfp* transcript when fused to the indicated *esxB* 5′ UTR variants were measured by quantitative PCR. The mean and 95% CI of triplicate datasets are shown. The upper error bar for the ∆hairpin 1 sample was clipped for visualization purposes. The differences between strains were not statistically significant (linear regression). (**D**) YFP protein abundance from transcripts with the indicated 5′ UTRs was measured by flow cytometry. A promoterless *yfp* gene was used as a control for autofluorescence. The median fluorescence from each of three biological replicates was determined and the mean and SD of those medians are shown. The means were compared by ANOVA, followed by Dunnett’s multiple comparisons test to compare “Full UTR” to each of the variants. ***, *p* < 0.001; **, *p* < 0.01; ns, *p* > 0.05.

All *M. smegmatis* strains were derived from mc^2^155 and are described in Table S1. The data in Figure 1 were generated from a derivative with the *hyg* resistance gene inserted upstream of, and divergent from, the *rne* gene as previously described [15]. This strain was created as a control and does not have altered *rne* expression [15]. The data in Figure 2, Figure 3 and Figure 4 were obtained from a strain with a clean deletion of msmeg_0062-msmeg_0066, which was a gift from Keith Derbyshire, Todd Gray, and Joseph Wade. The deletion strain was transformed with the plasmids described in Table S1. These plasmids are episomal and derived from pMV762. The promoter in pMV762 was deleted and replaced with msmeg_0063-0066 together with the promoter and 5′ UTR upstream of msmeg_0063 (227 nt upstream of the msmeg_0063 coding sequence), with the tsynA transcriptional terminator [21] placed upstream of the promoter to prevent read-through transcription from any spurious promoters present in the plasmid backbone. The data in Figure 5 were obtained with a wildtype strain transformed with hygR-marked Giles-integrating plasmids in which the Pmyc1-tetO promoter ([22], without its associated 5′ UTR) was linked to the 70 nt upstream of the *esxB* coding sequence and the *yfp* coding sequence. The tsynA transcriptional terminator [21] was placed immediately upstream of the promoter. All plasmids were constructed with a New England Biolabs HiFi assembly kit.

Liquid cultures were grown in Middlebrook 7H9 broth supplemented with 0.2% glycerol, 0.05% Tween 80, and Albumin Dextrose Catalase (ADC) to have final concentrations of 5 g/L bovine serum albumin fraction V (BSA), 2 g/L dextrose, 0.85 g/L sodium chloride, and 3 mg/L catalase. A final concentration of 250 μg/mL of hygromycin was used as needed. Solid cultures were grown on 7H10 with the same supplements except that glycerol was increased to 0.5% and Tween was omitted.

### 2.2. Flow Cytometry

Triplicate *M. smegmatis* cultures were grown to an optical density (OD) of 0.6 at 600 nm in freshly filtered media to reduce precipitates. The cultures were chilled on ice, diluted to an OD of 0.015 with freshly filtered 7H9, and filtered through a 5 μm filter needle to remove clumps. The samples were analyzed on a CytoFLEX flow cytometer (Beckman Coulter Life Sciences, Brea, CA, USA). For each sample, 60,000 events were collected, and a small gate was drawn around the densest region in a side scatter vs. forward scatter plot to select populations of primarily single cells for comparisons between strains.

### 2.3. RNA Extraction

Cultures were grown to an OD600 between 0.4 and 0.6 for the RNA extractions that were used for steady-state transcript abundance measurements. The culture pellets were frozen in liquid nitrogen and later thawed in 1 mL TriZOL (Thermo Fisher, Waltham, MA, USA) and lysed in tubes pre-filled with 600 mg of molecular-grade 100-micron Zirconium Beads (OPS Diagnostics, Readington, NJ, USA), using a FastPrep 5G (MP Biomedicals, Santa Ana, CA, USA) at 7 m/s for 30 s for a total of 3 cycles, with 2 min on ice between cycles. RNA was then extracted with a Direct-zol RNA extraction and purification kit (Zymo Research, Irvine, CA, USA) including on-column DNase treatment and eluted in H_2_O.

### 2.4. cDNA Synthesis

RNA (600 ng for qPCR and 300–477 ng for 5′ RACE) was diluted to 5.25 μL before 0.83 μL of 100 mM Tris pH 7.5 and 0.17 μL of 3 mg/mL random hexamer primers were added, and the samples were incubated at 70 °C for 10 min, then snap-cooled for 5 min in ice water. Reverse transcription was performed by adding 2 μL of 5X ProtoScript II Buffer, 0.5 μL of 10 mM each dNTP, 0.5 μL of 100 mM DTT, 0.25 μL of 40,000 U/mL RNase Inhibitor Murine (NEB, Ipswich, MA, USA), and 0.5 μL of 200,000 U/mL ProtoScript II Reverse Transcriptase (NEB, Ipswich, MA, USA) to the template–primer mix on ice. Equivalent reactions were also performed with an equal volume of H_2_O in place of the reverse transcriptase, to control for the presence of genomic DNA (no-RT controls). Reactions were incubated at 25 °C for 10 min followed by 42 °C for at least 5 h. Alkaline degradation was then performed to remove template RNA; 5 μL of 0.5 mM EDTA and 5 μL of 1 N NaOH were added to each sample, and this was followed by incubation for 15 min at 65 °C. Reactions were stopped with 12.5 μL of 1 M Tris HCl, pH of 7.5. cDNA cleanup was carried out with the Monarch^®^ PCR & DNA Cleanup Kit (5 μg) (NEB, Ipswich, MA, USA) according to the manufacturer’s directions, and the samples were eluted in H_2_O.

### 2.5. Quantitative PCR

cDNA samples were diluted to 1 ng/μL using RNase-free H_2_O and further diluted to bring the final concentration to 200 pg/μL. For each no-RT control, H_2_O was added in volumes equal to the corresponding cDNA sample. Each qPCR reaction contained 400 pg of cDNA or an equivalent volume of no-RT control, 1 μL of a solution with 2.5 μM of each appropriate primer, and 5 μL of iTaq Universal SYBR Green Supermix (BioRad, Hercules, CA, USA) in a total volume of 10 μL. Reactions were performed in 96-well plates. The plate was covered with a clear film and amplified with an Applied Biosystems 7500 qPCR machine. The samples were incubated at 50 °C for 2 min, at 95 °C for 10 min, and then for 40 cycles of 95 °C for 15 s followed by 61 °C for 1 min. The expression relative to *sigA* was defined as 2^−Δct^. The primers used for qPCR are listed in Table S2.

### 2.6. Rapid Amplification of cDNA Ends (RACE)

RNA samples were treated with high-concentration RNA Pyrophosphohydrolase (RppH) (NEB, Ipswich, MA, USA) according to manufacturer’s instructions to convert 5′ di- and triphosphates to monophosphates or were subjected to a mock treatment with H_2_O instead of RppH. The samples were then purified using the RNA Clean & Concentrator™-5 kit (Zymo Research, Irvine, CA, USA) according to manufacturer’s instructions with an elution in H_2_O. Next, a universal adaptor was ligated to RNA 5′ ends. A total of 510–721 ng of RNA in 8 µL was incubated with 1 µg of the oligo SSS1016 (see Table S2 for sequence) at 60 °C for 10 min and then snap-cooled on ice. The following were then added to each reaction: 10 µL of 50% PEG8000, 3 µL of 10X T4 RNA Ligase I Buffer (NEB, Ipswich, MA, USA), 3 µL of 10 mM ATP, 3 µL of dimethyl sulfoxide (DMSO), 1 µL of RNase Inhibitor Murine (NEB, Ipswich, MA, USA), and 1 µL of T4 RNA Ligase I (NEB, Ipswich, MA, USA). The reactions were incubated at 20 °C overnight and then purified using the RNA Clean & Concentrator™-5 kit (Zymo Research, Irvine, CA, USA) according to manufacturer’s instructions with an elution in H_2_O. This RNA was then used as a template for cDNA synthesis as described above and then subjected to PCR with an adapter primer and a gene-specific primer.

PCR reactions were performed in a total volume of 25 µL containing 2.5 µL 10X Taq Reaction Buffer (NEB, Ipswich, MA, USA), 1.25 µL 10 µM primer SSS101, 1.25 µL 10 µM reverse primer (designed for the gene of interest), 0.5 µL dNTP (10 mM each), 0.167 µL Taq DNA polymerase (NEB, Ipswich, MA, USA), 1.5 µL 2.25 ng/µL template cDNA with adaptor, and the remaining volume of H_2_O. Oligonucleotide sequences are shown in Table S2. The PCR conditions were as follows: (i) an initial step for DNA denaturing at 95 °C for 5 min, (ii) 35 cycles of 95 °C for 30 s, 52–57 °C for 20 s (depending upon the primer set), and 68 °C for 12 s, and (iii) a final elongation at 68 °C for 5 min. The PCR products were run on 2.0% agarose gels, and bands of interest were sliced from the gel and purified using the Zymoclean™ Gel DNA Recovery Kit according to the manufacturer’s instructions with an elution in H_2_O before being subjected to Sanger sequencing with the gene-specific primer.

### 2.7. mRNA Half-Life Measurement

*M. smegmatis* was grown to an OD_600_ of 0.8 in triplicate 50 mL cultures. In a 37 °C warm room, each culture was divided into five 8 mL cultures and placed on a culture wheel for 30 min. Rifampin was added to the cultures at a final concentration of 150 μg/mL, and they were frozen in liquid nitrogen after 0 s, 30 s, 1 min, 2 min, or 4 min. RNA extraction, cDNA synthesis and cleanup, and qPCR were performed as described above using primers SSS833 and SSS834 to amplify *yfp* [23], except that the cultures were pelleted after freezing in liquid nitrogen and thawing rather than before freezing. -C_T_ was used as a log_2_ metric of transcript abundance. When -C_T_ from all timepoints were plotted, it was evident that there was a delay in transcript degradation between 0 and 0.5 min and a plateau in degradation between 2 and 4 min. These trends have been described by us previously for many *M. smegmatis* genes [15]. We therefore used the 0.5–2 min timepoints to calculate half-life by linear regression.

### 2.8. Statistics

Graphpad Prism version 10.3.0 was used for all statistical analyses.

## 3. Results

### 3.1. The PE35_ms_-PPE68_ms_-esxB-esxA Locus in M. smegmatis Is Bisected by a Cleavage Site, and the esxB-esxA Portion Is More Abundant and More Stable than the Upstream Portion

Given the importance of *esxB* and *esxA* in *M. tuberculosis* virulence and *M. smegmatis* conjugation, we sought to better understand the determinants of their expression. We therefore examined the promoter landscape upstream of these genes in an *M. smegmatis* transcription start site (TSS)-mapping dataset [20]. These genes appeared to be transcribed from at least two promoters, with one TSS located at position −139 relative to the start codon of *msmeg_0063* and the other located at position −74 relative to the start codon of *esxB* (*msmeg_0065*) (Figure 1A, [20]). *msmeg_0063* and *msmeg_0064* are homologous to the *PE35* and *PPE68* genes of *M. tuberculosis*, which lie upstream of *esxB* and *esxA* in that organism and appeared to be co-transcribed in our previous study [15]. We therefore refer to *msmeg_0063* and *msmeg_0064* henceforth as *PE35_ms_* and *PPE68_ms_*, respectively. We also previously mapped two TSSs within the *esxB* coding sequence that could affect *esxA* expression but elected not to focus on them in the current study. The expression of *esxB* and *esxA* was substantially higher than that of *PE35_ms_* and *PPE68_ms_* (Figure 1A). There was an mRNA cleavage site mapped to the intergenic region between *PPE68_ms_* and *esxB* [20], four nucleotides (nt) downstream of a predicted TSS (Figure 1A). We therefore considered two non-exclusive hypotheses: (i) *esxB* and *esxA* have higher expression because of the strength of the promoter between *PPE68_ms_* and *esxB*, and (ii) the cleavage of transcripts made from one or both promoters results in a cleaved *esxB-esxA* transcript with greater stability. We then measured the half-lives of transcripts bearing *PE35_ms_*, *PPE68_ms_*, *esxB*, and *esxA*. The half-lives of the *PE35_ms_* and *PPE68_ms_* transcripts (2.5 and 3.0 min, respectively) were substantially shorter than those of the *esxB* and *esxA* transcripts (4.9 and 5.4 min, respectively; Figure 1B). This result is consistent with either hypothesis.

### 3.2. At Least Two Promoters Contribute to esxB and esxA Expression in M. smegmatis

To test the roles of the promoters upstream of *PE35_ms_* and *esxB*, we obtained an *M. smegmatis* strain with an unmarked deletion of the putative *PE35_ms_*-*PPE68_ms_-esxB-esxA* operon, as well as the upstream gene *msmeg_0062*, and transformed it with a series of plasmids containing *PE35_ms_*-*PPE68_ms_-esxB-esxA* with a deletion of the promoter upstream of *PE35_ms_* (TSS_1_) as well as mutations to the putative −10 site upstream of *esxB* (TSS_2_; Figure 2A). We then utilized quantitative polymerase chain reaction (qPCR) to measure the abundances of *PPE68_ms_*, *esxB*, *esxA*, and a region spanning both TSS_2_ and the cleavage site between *PPE68_ms_* and *esxB* (Figure 2B). We elected not to include *PE35_ms_* in these experiments because its abundance and half-life were similar to those of *PPE68_ms_* (Figure 1) and because we did not detect putative TSSs or cleavage sites between *PE35_ms_* and *PPE68_ms_* [15,20]. We found that the expression of *PPE68_ms_* was nearly eliminated when the upstream promoter corresponding to TSS_1_ was deleted, consistent with the *PPE68_ms_* transcript being produced solely by that promoter. In contrast, the abundances of the *esxB* and *esxA* transcripts were reduced but still substantial. The mutation of the putative promoter shortly upstream of *esxB* (corresponding to TSS_2_) caused only a modest decrease in the abundances of *esxB* and *esxA*. The disruption of both promoters simultaneously led to dramatically reduced abundances of *esxB* and *esxA*. This suggests that both promoters contribute to the expression of *esxB* and *esxA* in a generally additive fashion, with the first promoter having a somewhat larger role. Some expression remained even when those two promoters were disrupted, suggesting that yet another promoter may be contributing to expression of *esxB* and *esxA*. The *esxA* expression could potentially be explained by putative TSSs previously identified in the *esxB* coding sequence [20], but the location of the *esxB* primers is upstream of these TSSs and is therefore not explained. We were unable to identify a 5′ end corresponding to the TSS from this putative third promoter upstream of *esxB*.

To confirm the positions of TSS_1_ and TSS_2_, we performed 5′ Rapid Amplification of cDNA Ends (RACE). TSS_1_ was easily confirmed at the position indicated in our transcriptome-wide study, −139 relative to the *PE35_ms_* start codon. The verification of TSS_2_ was more challenging because the RNA cleavage site produces ample 5′ ends that are readily ligatable, making the detection of other lower-abundance 5′ ends difficult (Figure 3A). By analyzing 5′ RACE sequencing traces with mixed peaks, we were able to detect 5′ ends at positions 3 and 4 nt upstream of the cleavage site (Figure 3B). These are located 8 and 7 nt downstream of the −10 site, respectively, which corresponds to the expected spacing between mycobacterial −10 sites and TSSs [13,14,20].

### 3.3. mRNA Cleavage Can Be Reprogramed by a Single Point Mutation but Is Not Required for Normal Levels of esxB and esxA Expression in M. smegmatis

The apparently high proportion of the *esxB-esxA* transcript that was cleaved, the partial dependence of those transcripts on the upstream promoter for expression, and their longer half-lives compared to *PE35_ms_*-*PPE68_ms_* led us to question if cleavage affected the abundance of transcripts arising from the *PE35_ms_*-*PPE68_ms_-esxB-esxA* locus. The cleavage site had a cytidine at the +1 position, which we previously found to be true of most *M. smegmatis* and *M. tuberculosis* mRNA cleavage sites and is a determinant of cleavage by mycobacterial RNase E [15,20]. We therefore mutated the +1 cytidine to guanosine in our *PE35_ms_*-*PPE68_ms_-esxB-esxA* construct, alone and in combination with promoter mutations (Figure 4A). We then performed 5′ RACE to assess the impact of the mutation on mRNA cleavage (Figure 4B). In constructs with the C→G mutation, no 5′ ends were mapped at the original cleavage site. Instead, new 5′ ends were identified at positions −1 and −28 relative to the original cleavage site. The −28 cleavage event occurred with a cytidine at the +1 position, consistent with the typical mycobacterial cleavage signature, while the −1 cleavage event occurred with an adenosine at the +1 position, which differed from the typical mycobacterial cleavage signature. This result suggests that the mutation prevented cleavage at the original site but that other sequence and/or secondary structure features conducive to cleavage remained, resulting in cleavage at nearby positions.

To assess the impact of the cleavage site mutation on transcript abundance, we measured abundance for *PPE68_ms_*, *esxB*, *esxA*, and the junction region by qPCR (Figure 4C). Despite the impact of the cleavage site mutation on transcript 5′ end positions, it did not cause substantial or reproducible changes in the abundance of the measured transcript regions. This suggests that the precise position of the transcript 5′ end does not have a major impact on half-life.

### 3.4. The Stability of the M. smegmatis esxB-esxA Transcript Is Not Dependent upon 5′ End Secondary Structure

We noted the presence of a pair of strong hairpins in the predicted secondary structure near the 5′ end of the *esxB-esxA* transcripts that result from transcription at TSS_2_ and/or cleavage (Figure 5A). Given that secondary structure near transcript 5′ ends has been reported to stabilize transcripts in some bacteria [24,25,26,27,28,29,30,31,32,33], we hypothesized that these hairpins may contribute to the longer half-lives of *esxB* and *esxA* transcripts compared to *PE35_ms_* and *PPE68_ms_* transcripts (Figure 1B). To test this idea, we designed reporter constructs in which the 70 nt *esxB* 5′ UTR resulting from cleavage was fused to sequence encoding a previously described Yellow Fluorescent Protein (YFP) variant [23]. The reporter transcript was expressed from the Pmyc1-tetO promoter [22], which was constitutively active since the plasmids were integrated into a strain that did not express a Tet repressor. We then constructed variants in which the first hairpin (nt 1–23), second hairpin (nt 25–44), or both hairpins (nt 1–44) were deleted. These deletions were not predicted to affect ribosome binding, as the deleted regions were located upstream of the ribosome binding region. qPCR revealed that the deletion of either hairpin individually did not affect steady-state abundance of the *yfp* transcript, while the deletion of both hairpins produced higher transcript levels (Figure 5B). This result suggested that the hairpins did not increase the stability of the transcript. To test this, we measured the half-lives of the *yfp* transcript fused to each 5′ UTR variant and found that the half-lives were not distinguishable (Figure 5C). We therefore conclude that, at least when fused to *yfp*, the secondary structure of the *esxB* 5′ UTR does not increase the stability of the transcript. Consistent with the observed differences in mRNA abundance, we found that the levels of YFP protein were the highest when expressed on a transcript with the UTR deleted for both hairpins, and the full-length UTR and single hairpin deletions all displayed similar YFP protein levels to each other (Figure 5D).

### 3.5. Secondary Structure near the 5′ Ends of 5′ UTRs Is Not Generally Correlated with mRNA Stability in M. smegmatis

**Figure 6:**
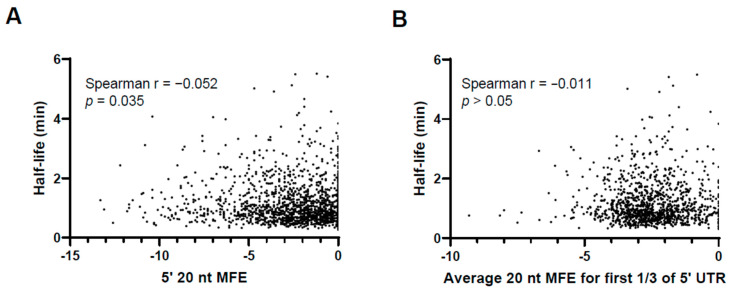
The extent of secondary structure near the 5′ ends of 5′ UTRs does not generally correlate with transcript stability. The half-lives of ~1800 leadered *M. smegmatis* genes were previously reported and plotted here as a function of two metrics of 5′ UTR secondary structure [34]. Relationships were assessed by Spearman’s correlation. (**A**) The 20 nt at the 5′ end of each 5′ UTR was computationally folded by RNAfold (Vienna RNA Package) as described [34]. The minimum free energy (MFE) of the MFE structure for each 5′ UTR is plotted. (**B**) The 5′ third of each 5′ UTR was computationally folded in 20 nt windows with 10 nt overlaps using RNAfold (Vienna RNA Package) as described [34]. For each 5′ UTR, the mean MFE of the resulting structures is plotted.

To assess the impact of secondary structure near the 5′ ends of 5′ UTRs on mRNA stability in *M. smegmatis*, we examined the relationship between predicted 5′ UTR secondary structure and mRNA half-life from a previously published transcriptome-wide half-life dataset [15,34]. In a previous study, we determined the half-lives of the transcripts of several thousand *M. smegmatis* genes during log-phase growth [15]. Here, we focused on the ~1800 transcripts within that dataset that are known to have 5′ UTRs [20]. For these transcripts with defined 5′ UTRs, we previously examined two metrics of secondary structure near the 5′ ends of the 5′ UTRs [34]. First, we computationally folded the first 20 nt of each transcript to predict the minimum free energy (MFE) structure [34]. Here, we assessed the correlation between the free energy of each structure and the half-life of the corresponding transcript and found only a very weak correlation that, while statistically significant, is unlikely to be biologically significant (Figure 6A). Since secondary structure may involve more than the first 20 nt of a transcript, we also predicted the MFE structures for sliding 20 nt windows within the first third of each 5′ UTR [34]. Here, we examined the correlation between the average free energy for the sliding windows within the first third of each 5′ UTR and the half-life of the corresponding transcript (Figure 6B). We did not observe a statistically significant correlation. Together, these results suggest that secondary structure near the 5′ ends of 5′ UTRs is not a major determinant of mRNA stability in *M. smegmatis*.

## 4. Discussion and Conclusions

Here, we have examined the presence and impact of TSSs and RNA cleavage sites in an operon encoding key components of the ESX-1 secretion system. We found that at least two separate promoters contribute to the transcription of *esxA* and *esxB*: one located immediately upstream of *esxB* and another located upstream of *PE35_ms_* and presumably producing a four-gene polycistron. One or both of these transcripts is cleaved 4 nt downstream of the *esxB*-proximal TSS, and *esxB* and *esxA* have longer mRNA half-lives than *PE35_ms_* and *PPE68_ms_*. The mutation of a single nt at the cleavage site changes the position of cleavage but does not affect the steady-state abundance of the *esxB-esxA* transcript. Finally, we assessed the impact of two hairpin structures predicted to be formed by the *esxB* 5′ UTR and found that, contrary to expectations, these structures did not protect a reporter transcript from degradation. Consistent with this, we were unable to detect a relationship between 5′ UTR secondary structure and mRNA degradation rate transcriptome-wide, suggesting that 5′ UTR secondary structure may not be a major determinant of mRNA stability in mycobacteria.

Previous studies have identified transcription factors involved in the regulation of ESX-1 genes in *M. tuberculosis* and *M. marinum*. In *M. tuberculosis*, the expression of *esxB-esxA* is regulated by PhoP, WhiB6, and EspR [12,35,36], although only WhiB6 appears to be a direct regulator of *esxB-esxA* transcription, likely by binding at the promoter upstream of *PE35* [11]. The role of WhiB6 in regulation of *esxB-esxA* has been shown to be conserved in *M. marinum* [37,38], and the conserved transcription factors EspM and EspN have been shown to be regulators of *whiB6* and possibly other ESX-1 genes in that organism [39,40]. Additional transcription factors have been reported to bind to promoters in the *PE35-PPE68-esxB-esxA* region in *M. tuberculosis*, but most of their roles have not been experimentally tested [11]. To our knowledge, the transcriptional regulation of *esxB-esxA* has not been studied in *M. smegmatis*. Further work is therefore needed to determine the significance of the two (or more) promoters driving the expression of these genes.

Our results leave open the question of the role of the prominent RNA cleavage site between *PPE68* and *esxB*. This cleavage site is conserved in *M. tuberculosis* [15], suggesting that it is functionally significant, yet mutation of the cleavage site in *M. smegmatis* did not affect the abundance of the corresponding transcripts. It is possible that the mutation did not affect transcript abundance because cleavage still occurred, albeit at different locations. Our methodology does not permit a quantitative assessment of the relative amount of cleavage in the wildtype transcript vs. that with the cleavage site mutation. It is also possible that the cleavage event could affect transcript stability in conditions other than the single log-phase growth condition that we assessed in this work.

Given our previous finding that RNase E specifically cleaves the phosphodiester linkage immediately upstream of cytidines, it was surprising to observe that, in the cleavage site mutant, detectable cleavage occurred between two adenosines. This suggests that RNase E is capable of cleaving non-preferred nucleotide sequences and that its cleavage is directed by additional sequence or secondary-structure features besides the sequence in the immediate vicinity of the cleaved bond. For example, secondary structure elements near single-stranded cleavage sites have been suggested to facilitate cleavage by *E. coli* RNase E [41]. Further work is needed to identify these other determinants of RNase E activity in mycobacteria.

Previous reports have indicated that 5′ end secondary structure protects specific transcripts from degradation in various bacteria [24,25,26,27,28,29,30,31,32,33], and this is consistent with the idea that some RNases with key roles in degradation, such as RNase E in *E. coli* and mycobacteria and RNase J in *Bacillus subtilis*, directly engage transcript 5′ ends [42,43,44,45,46,47,48]. Mycobacteria encode both RNase E, which has a rate-limiting step in the degradation of most mRNAs [15], and RNase J, which has a specialized role affecting fewer transcripts [49]. However, the results presented here indicate that secondary structure at or near the 5′ ends of 5′ UTRs is not a major contributor to mRNA half-life and does not impact the half-life of the specific transcript we tested. These results do not necessarily indicate fundamental differences between mycobacteria and other species for which 5′ end secondary structure has been shown to be protective; rather, it is possible that 5′ end secondary structure protects some transcripts in mycobacteria but that this effect is not widespread enough to be seen on a transcriptome-wide scale. This is consistent with our recent findings that mRNA half-lives in *M. smegmatis* are determined by the complex interplay between a number of transcript characteristics, with no particular transcript characteristic having a dominant role on a transcriptome-wide scale [34]. It should also be noted that the half-lives of the native *esxB* and *esxA* transcripts were substantially longer (~5 min) than those of the *yfp* reporter with the *esxB* 5′ UTR (~30 s). This indicates that other features of the transcripts have major impacts on their degradation rates.

The results described here contribute to a growing foundation of knowledge about how mycobacteria regulate genes important for phenotypes of interest such as virulence and the generation of genetic diversity. Further work will be needed to determine how ESX-1 promoter structure differs among mycobacterial species as well as the roles of transcription factors in regulating expression from the promoters identified.

## Data Availability

The RNAseq data used to create Figure 1 can be accessed on GEO, accession number GSE227248.

## Supplementary Materials

The following supporting information can be downloaded at https://www.mdpi.com/article/10.3390/pathogens13121041/s1, Table S1. Strains and plasmids used in this work. Table S2. Oligonucleotides used in this work.
