# Supplementary material for: Functional Analysis of Promoters, mRNA Cleavage, and mRNA Secondary Structure on esxB-esxA in Mycolicibacterium smegmatis
Source: Pathogens. 2024 Nov 27;13(12):1041. doi: 10.3390/pathogens13121041 (PMC11728522; doi:10.3390/pathogens13121041)
Supplement: Supplementary file 1 [file pathogens-13-01041-s001.zip › Table S2.pdf]

Table S2. Oligonucleotides used in this work. The description and sequence of all oligonucleotides used throughout the study. Letters preceded by a lowercase "r" are ribonucleic acids, while all others are deoxyribonucleic acids. 'Fw' denotes a forward primer, and 'Rv' denotes a reverse primer.

**Description**	**Name**	**Sequence (5' -> 3')**
Fw *PPE68* qPCR primer	SSS545	TTTCAGCAGCGAGAGTTAGG
Rv *PPE68* qPCR primer	SSS546	GTGTTGATCTCCGGTGGTAG
Fw Spanning cleavage site qPCR primer	SSS549	GACCTCTCCGAGGATGAAGA
Rv Spanning cleavage site qPCR primer	SSS550	CTGTGTCCTCACCAATTCCA
Fw e*sxB* qPCR primer	SSS537	GGTGAGGACACAGGGAAATAAG
Rv e*sxB* qPCR and 5' RACE primer for TSS_2 and cleavage site	SSS538	CGGAGATGCGCTCGAAAT
Fw *esxA* qPCR primer	SSS695	CTCCAACGAGCTGAACCT
Rv *esxA* qPCR primer	SSS696	GGCAAACATTCCCGTGAC
5' RACE adaptor	SSS1016	CTGGAGCACGAGGACACTGACATGGACTGAAG GAGTrArGrArArA
Fw adaptor primer for 5' RACE	SSS1017	CTGGAGCACGAGGACACTGA
Rv *PE35* 5' RACE primer for TSS_1	SSS987	GGATTGTGTGTCATCGGTTG
